# Hidden Markov models lead to higher resolution maps of mutation signature activity in cancer

**DOI:** 10.1186/s13073-019-0659-1

**Published:** 2019-07-26

**Authors:** Damian Wojtowicz, Itay Sason, Xiaoqing Huang, Yoo-Ah Kim, Mark D. M. Leiserson, Teresa M. Przytycka, Roded Sharan

**Affiliations:** 1 National Center for Biotechnology Information, National Library of Medicine, National Institutes of Health, 8600 Rockville Pike, Bethesda, 20894 USA; 2 Center for Bioinformatics and Computational Biology, University of Maryland, 8125 Paint Branch Dr, College Park, 20740 USA; 3 School of Computer Science, Tel Aviv University, Tel Aviv, 69978 Israel

**Keywords:** Mutational process, Hidden Markov model, Mutation signature, Breast cancer

## Abstract

Knowing the activity of the mutational processes shaping a cancer genome may provide insight into tumorigenesis and personalized therapy. It is thus important to characterize the signatures of active mutational processes in patients from their patterns of single base substitutions. However, mutational processes do not act uniformly on the genome, leading to statistical dependencies among neighboring mutations. To account for such dependencies, we develop the first sequence-dependent model, SigMa, for mutation signatures. We apply SigMa to characterize genomic and other factors that influence the activity of mutation signatures in breast cancer. We show that SigMa outperforms previous approaches, revealing novel insights on signature etiology. The source code for SigMa is publicly available at https://github.com/lrgr/sigma.

**Electronic supplementary material:**

The online version of this article (10.1186/s13073-019-0659-1) contains supplementary material, which is available to authorized users.

## Background

Cells acquire somatic mutations over time from exposure to different combinations of mutational processes, potentially leading to cancer. Understanding the activity of mutational processes is critical for cancer treatment, as many standard treatments introduce DNA damage or inhibit DNA damage repair genes [1, 2]. Presently, clinicians use specialized assays for specific biomarkers to characterize DNA damage repair deficiencies, such as microsatellite instability (see, e.g., [3]). Large-scale cancer sequencing efforts have recently opened up new avenues for characterizing the activity of mutational processes. The key insight is that mutational processes leave *signatures* of their activity in cancer genomes, the most well-studied of which are patterns of base substitutions.

An increasing body of research aims at inferring signatures and their exposures from large datasets of mutations from cancer whole-exome and whole-genome sequences [4–10], and the Catalogue of Somatic Mutations in Cancer (COSMIC) consortium has collected a census of 30 validated mutation signatures [11]. Many of these signatures are associated with deficient DNA damage repair pathways; some have been validated experimentally [12, 13], expanding the opportunity for targeted therapy. For example, Davies et al. [14] provided evidence that mutation signatures reveal patients deficient in homologous recombination repair (HR) and thus may benefit from PARP inhibitor treatment. Importantly, some of these patients do not harbor biallelic inactivations in known HR genes. Other signatures are associated with environmental exposures to carcinogens such as tobacco smoke [15] or aflatoxin [16], and two are associated with aging [17] indicating that the underlying mutational processes may be active in healthy cells.

Despite these advances, uncovering etiology of mutation signatures and inferring their exposures remain significant challenges, e.g., about half of the COSMIC signatures have no known etiology. Even with validated mutation signatures, it can be difficult to infer their exposures and assign individual mutations to the corresponding signature, in part because there may be multiple signatures of the same mutational process. One key factor to inferring signature exposure is the sequential dependency of the signatures. This is the idea that mutations that are adjacent in a given cancer genome are more likely to be the result of the same mutation signature. In their seminal work, Nik-Zainal et al. [18] identified clusters of mutations in breast cancers (termed *kataegis*) that display a particular base substitution signature. Kasar et al. [7] uncovered a signature of “canonical” activation-induced cytidine deaminase (AID) pathway activity in chronic lymphocytic leukemia that was missed by Alexandrov et al. [4]. Part of the reason for their discovery was that they incorporated the “nearest mutation distance” into their model, since AID is known to cause multiple mutations within local regions of the genome. Morganella et al. [19] identified the so-called processive groups of up to 20 mutations believed to come from the same signature. Morganella et al. [19] and Haradhvala et al. [20] both characterized signatures in terms of the transcriptional and replicative strands and replication timing. Supek and Lehner [21] identified mutation signatures that are specifically associated with clusters of mutations and showed that the activity of these signatures is associated with an increase in the mutation rate of expressed genes.

Motivated by this earlier work, we set out to model the genomic factors that bias mutational process activity, such as genome position, CpG islands, and replication origins. We hypothesized that by capturing the statistical dependencies introduced by these genomic factors, our models would yield more precise estimates of mutation signature exposure, and would further reveal genomic features that correlate with mutational process activities. Our contribution is threefold: (i) we suggest the first probabilistic model to account for sequential dependency among mutation signatures; (ii) we use this model to rigorously assign mutation signatures to individual mutations and characterize the genomic and phenotypic preferences of mutation signatures; and (iii) we study the transition probabilities between different mutation signatures.

## Methods

### A hidden Markov model of mutation signatures

Following previous work, we categorize mutations in a cancer genome into *L*=96 categories that include its base substitution (C:G >A:T, C:G >T:A, C:G >G:C, A:T >C:G, A:T >T:A, A:T >G:C), and left- (4) and right-flanking (4) nucleotides [4]. We model an observed sequence of mutations using a hidden Markov model (HMM). The model assumes that each observation, representing a mutation category, is emitted by one of *K* states in a Markov chain, representing a mutation signature. The sequence of states that generated the observed sequence is unknown, but as the states form a Markov chain, each state depends on the previous state, thus capturing sequential dependencies between states. An HMM is parameterized by a vector *π* of *K* starting probabilities, a *K*×*K* transition matrix *A*, and a *K*×*L* emission matrix *E*.

The above HMM can capture sequential dependencies but is less motivated for “isolated” mutations that are distant from any other mutation. We call such distal mutation regions *sky* and refer to regions of proximal mutations as *clouds* (using a distance threshold of 2000 bp as explained below).

**Fig. 1:**
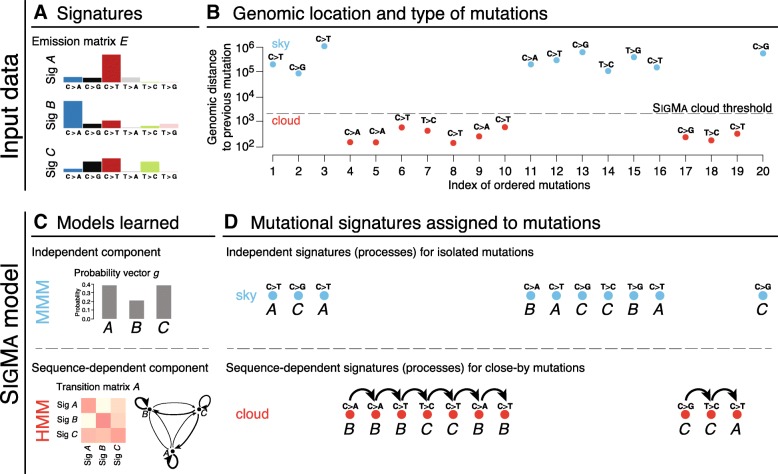
Overview of the SIGMA model. The input data consists of (**a**) a set of predefined signatures that form an emission matrix *E* (here, for simplicity, represented over six mutation types) and (**b**) a sequence of mutation categories from a single sample and a distance threshold separating sky and cloud mutation segments. **c** The SIGMA model has two components: (top) a multinomial mixture model (MMM) for isolated sky mutations and (bottom) an extension of a hidden Markov model (HMM) capturing sequential dependencies between close-by cloud mutations; all model parameters are learned from the input data in an unsupervised manner. **d**SIGMA finds the most likely sequence of signatures that explains the observed mutations in sky and clouds

We model sky mutations using a multinomial mixture model (MMM). The MMM is characterized by a vector *g* of *K* mutation signature marginal probabilities and the same emission matrix *E*. To model cloud mutations, we use a dynamic Bayesian network (DBN) that is a simple extension of an HMM in that it allows subsequences generated by the HMM to be interspersed with mutations generated by the MMM (for a review of DBNs, see [22]). We call the resulting composite model Signature Markov model (SIGMA); a simplified overview of the model is presented in Fig. 1, and its cloud component is sketched in Additional file 1: Figure S1.

We now define the SIGMA model for clouds. The input data is a sequence of $O_{1}, \dots, O_{T}$ mutation categories. The (hidden) signature that generated mutation category *O*_*t*_ is represented by *Q*_*t*_. The transitions between signatures at each subsequent position depend on whether the observed mutation category occurs within sky (marked by a binary indicator *I*_*t*_) or clouds. The joint probability distribution of the model is:

1 $${} {\begin{aligned} &\Pr(O_{t=1}^{T}, Q_{t=1}^{T}, I_{t=1}^{T})\\ &\quad= \Pr(Q_{1})\Pr(I_{1})\left[\prod\limits_{t=2}^{T} \Pr(Q_{t} | Q_{t-1}, I_{t-1}) \Pr(I_{t}|I_{t-1})\right]\prod\limits_{t=1}^{T}\Pr(O_{t}|Q_{t}). \end{aligned}}  $$

We now define the conditional probability distributions (CPDs). The transition between signature states *Q*_*t*−1_ to *Q*_*t*_ depends on the indicator *I*_*t*−1_ in the following manner. Within sky, the transitions occur according to the marginal probability of each state (i.e., as in the MMM), while otherwise the transitions to state *Q*_*t*_ depend on state *Q*_*t*−1_. Formally, when *I*_*t*_=0 (i.e., the current mutation is in a cloud):

2 $$ \Pr(Q_{t}=j | Q_{t-1}=i, I_{t-1}=f) = \left\{\begin{array}{ll} A_{ij} & \text{if}\ f = 0, \\ \pi_{j} & \text{if}\ f = 1. \end{array}\right.  $$

The probability of the initial state depends only on the starting probabilities of the signatures, such that $\Pr (Q_{1}=i) = \pi _{i}$.

The transitions between the sky segment indicator *I*_*t*_ only depends on the previous indicator *I*_*t*−1_, i.e.,

3 $$ \Pr(I_{t}=j|I_{t-1}=i) = B_{ij},  $$

where *B* is the 2×2 transition matrix between the sky and cloud segments. Note that *B* implicitly governs the length of those segments and can be learned directly from observed data. The probability of starting in a sky/cloud state is given by $\Pr (I_{1}=i)=\rho _{i}$, where *ρ* is a 2×1 starting probability vector. Finally, given the state *Q*_*t*_, each observation *O*_*t*_ is independent of all other variables, i.e.,

4 $$ \Pr(O_{t}=j | Q_{t}=i) = E_{ij}.  $$

### Model training

We learn the SIGMA model parameters from data using the Baum-Welch expectation-maximization algorithm with random initialization. We then compute Viterbi paths—the most likely sequence of states that generated the data—to assign mutations to signatures and compute signature *exposures* (i.e., signature frequency per sample). In practice, we find that the assignments are robust with respect to the random initialization used in the learning process; on average, over 95% of mutations are assigned to the same signature when compared to the majority assignments in 31 random initialization runs of SIGMA, and the standard errors of the presented results are small with respect to the random initializations.

Rather than model the mutations in a cohort of cancer genomes with a single SIGMA, we train a model per sample. The motivation for this approach comes from the assumptions of earlier methods (e.g., [5]) that signature exposures are different across samples.

**Fig. 2:**
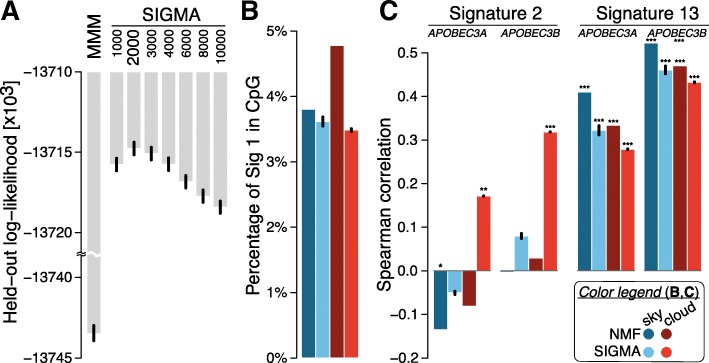
**a** Comparative assessment of model performance on held-out data for MMM and SIGMA across different distance thresholds. SIGMA at a threshold of 2000 bp shows the best performance by maximizing the log-likelihood (the *y*-axis has a customized scale with a scale break). **b** Comparison of fraction of signature 1 mutations found in CpG islands in sky and clouds. Both NMF and SIGMA show significant depletion of signature 1 in CpG islands with respect to randomized data, with SIGMA exhibiting more pronounced depletions, particularly in clouds. We performed 1000 permutations of signature assignments preserving mutation trinucleotide context within each sample. We used a one-sided Wilcoxon signed-rank test to compare the observed and randomized numbers of signature 1 in CpG islands. **c** Spearman correlation comparison of *APOBEC3A/B* expression with signature 2 and 13 activities across samples. For signature 2, the mutation counts in clouds with SIGMA are positively correlated with *APOBEC3A/B* expression while the NMF-based counts have zero or negative correlation in both sky and clouds. Signature 13 mutation counts are positively correlated in both models. In **b** and **c**, the significance level was categorized as *P value (*P*) < 0.05; ** *P*<5×10^−3^; *** *P*<5×10^−5^. All bar plots show mean values with standard error of the mean (small black bars) from 31 random initializations of MMM and SIGMA models

The SIGMA model has several meta-parameters that are set in advance: (i) the set of signatures used and (ii) a distance threshold indicating the beginning of a new segment (cloud or sky) of mutations. In this work, we focus on the assignment of signatures to mutations rather than on signature learning; hence, we consider only COSMIC signatures [23], focusing on the signatures previously identified as active in breast cancers: signatures 1, 2, 3, 5, 6, 8, 13, 17, 18, 20, 26, and 30. For the other meta-parameter, we perform model selection and evaluate the performance of each choice using the log-likelihood of the model on held-out data. To this end, we use a leave-one-out cross-validation scheme leaving one of the chromosomes out. We report the median SIGMA held-out likelihood across the different initializations. The results are summarized in Fig. 2a, and accordingly, we set the distance threshold to 2000 bp at which the held-out log-likelihood was maximized. Thus, a mutation whose flanking mutations (if any) are more than 2000 bp away is called *sky*; otherwise, the mutation is considered to be within a *cloud*.

We note that while SIGMA always models the mutations in sky as being independent from one another, the Markovian component of SIGMA learns whether the mutations in clouds are sequence dependent or independent. In practice, approximately 71% of mutations in clouds are found to be sequence dependent according to the most likely sequence of mutation events.

### Software availability

We implemented SIGMA in Python 3. The code is publicly available at https://github.com/lrgr/sigma. On average, it takes approximately 8 seconds to train SIGMA on a single sample of breast cancer whole genome, learning a total of 146 model parameters.

### Data

We analyzed 3,479,652 mutations in the cohort of 560 breast cancer (BRCA) whole genomes previously analyzed by Nik-Zainal et al. [24]. Each patient has an average of 208.2 clouds containing an average of 2.33 mutations, with 271,492 total mutations in clouds (8*%*) and 3,208,160 total mutations in sky (92*%*).

We also analyzed single base substitutions from the International Cancer Genome Consortium Data Portal [25] in 160 pancreatic cancer (PACA), 151 chronic lymphocytic leukemia (CLLE), and 241 malignant lymphoma (MALY) whole-cancer-genome sequences. The PACA data is from ICGC release 25 (PACA-AU), and we restricted to the sample per patient with the most mutations, and we removed patients where all samples had fewer than 500 mutations. We analyzed COSMIC signatures 1, 2, 3, 5, 6, and 13 in the PACA dataset. The CLLE and MALY data is from ICGC release 27 (CCLE-ES and MALY-DE) and is also restricted to the sample with the most mutations per patient. We analyzed COSMIC signatures 1, 2, 5, 9, and 13 in the CLLE dataset and COSMIC signatures 1, 2, 5, 9, 13, and 17 in the MALY dataset.

To compare SIGMA to NMF, we recomputed the NMF assignments of signatures to mutations used by Morganella et al. [19] following their maximum likelihood approach. We downloaded the gene expression data for 266 BRCA samples from Table S7 in Nik-Zainal et al. [24]. For replication timing analysis, we downloaded percentage normalized replication time estimates from Repli-seq data in the MCF-7 cell line from the ENCODE project [26], and we split them into deciles and counted sky and cloud mutations in each decile. All analyses related to replication time were corrected for genomic size by accounting for unknown (*N*) bases. The MCF-7 cell line was chosen as it most closely represents breast cancers (see Morganella et al. [19] for details). The CpG islands’ coordinates were downloaded from the UCSC Genome Browser [27] and gene annotations from the ENSEMBL database (release 60) [28].

We evaluated inferred signature assignments to mutations in part using clinical and demographic features of each of the 560 cancers. We downloaded clinical and demographic data from Table S1 in Nik-Zainal et al. [24], restricting our analysis to those features that are measured in at least 85% of the patients (omitting gender since the cohort is >99% female): age, tumor grade, estrogen-receptor (ER) status, progesterone-receptor (PR) status, and HER2 status. We imputed missing data using the mean.

## Results

In order to capture the sequential dependencies among mutation signatures, we propose a hidden Markov modeling framework. Within this framework, the identity of the mutation signature underlying a given mutation depends (through conditional probability) on the identity of the signature that yielded the preceding mutation in the genome. This modeling approach is motivated by earlier work that has shown that the mutations in localized clusters are often found to be from the same signatures [19, 21], thus suggesting a sequential dependency among mutation signatures. However, the majority of mutations in the cancer genome are hundreds of thousands of base pairs from the nearest mutation, suggesting that this dependency only manifests on small, localized regions of the cancer genome. To account for this complexity, we develop a composite model, SIGMA, that can infer the sequential dependencies among mutation signatures within localized densely mutated regions. We train our model and apply it to 560 breast cancer whole genomes previously analyzed in [24], partitioning each tumor’s mutation into *sky* (isolated mutations) and *clouds* (groups of close-by mutations). The model is sketched in Fig. 1; full details on the model, its training, mutation partitioning, and data appear in the “Methods” section.

### SIGMA uncovers sequential dependency between mutation signatures and leads to stronger associations with related biological signals

To assess the utility of SIGMA in capturing sequential dependencies, we compare it to a baseline probabilistic model with no sequential dependencies. Since the state-of-the-art method for inferring mutation signatures, non-negative matrix factorization (NMF), is non-probabilistic, we use a related multinomial mixture model (MMM) as our baseline. The model parameters are learned so as to maximize the likelihood of the model using expectation maximization. Both SIGMA and MMM were applied to each sample separately, fixing the 12 COSMIC signatures previously found to be active in breast cancer (see the “Methods” section for details).

Figure 2a summarizes the performance of the models in cross-validation on a breast cancer dataset of 560 genomes. We draw two conclusions from these results. First, there is a significant sequential dependency among the mutation signatures within clouds, as the variants of SIGMA all outperform the baseline MMM. Second, the sequential dependency is strongest for mutations within 1000–4000 bases of one another; SIGMA achieves the highest held-out log-likelihood in this range using a distance threshold of 2000 bp. Thus, we adopt the threshold of 2000 bp for the remainder of our experiments. Following previous studies that indicated that certain cloud-like regions are formed by a single mutational process [19], we also tested a variant of our model in which a single signature is allowed within a cloud, reflecting the hypothesis that each cloud was formed in a single sweep. SIGMA outperformed this variant in log-likelihood on held-out data (− 13,714,498 vs. − 13,725,785). It also outperformed a variant in which only APOBEC signatures 2 and 13 (potentially interspersed by sky mutations) are allowed within clouds (log-likelihood of − 13,718,394), supporting the greater flexibility our model allows within those regions. A summary of the performance of the models in cross-validation on additional cancer types is presented in Additional file 1: Figure S2, where it is shown that SIGMA outperforms the baseline MMM.

An important feature of our proposed probabilistic model is that it allows inferring the most likely mutation events that led to the observed data. Hence, we wished to assess if the inferred assignments of signatures to mutations can strengthen the associations with related biological signals in comparison with NMF-based assignments [19]. One of the best understood signatures is the age-related signature 1, the result of an endogenous mutational process initiated by spontaneous deamination of 5-methylcytosine. This process occurs at cytosine-guanine (CpG) dinucleotides and is related to the major site of cytosine methylation which carries the risk of spontaneous deamination of 5-methylcytosine (5mC) to yield thymine. CpG methylation is a silencing mark. CpG islands are GC-rich genomic regions that are often located around gene promoters of active genes and are typically not methylated [29, 30]. Thus, we expect a depletion of signature 1 in those regions after correction for trinucleotide context of mutations. While both models shows significant depletion of signature 1 in CpG islands, SIGMA exhibits more pronounced depletions, especially in clouds (Fig. 2b).

APOBEC enzymes are another relatively well-understood source of mutations in cancer. The APOBECs deaminate cytosines in single-stranded DNA, preferentially at TpC sequence context and are thus believed to be associated with signatures 2 and 13. In particular, *APOBEC3A* and *APOBEC3B* are among the main factors causing mutations in human cancers and specifically implicated in inducing clustered mutations (kataegis) [31–33], prompting us to test for an association between *APOBEC3A/B* expression and the number of mutations attributed to signatures 2 and 13. Surprisingly, the NMF-based mutation assignments show no or negative correlation between signature 2 and *APOBEC3A/B* expression both in sky and clouds, and a statistically significant correlation is observed only with signature 13. In contrast, using mutation assignments from SIGMA, we find that the signature 2 mutation counts in clouds show positive correlations with the *APOBEC3A* and *APOBEC3B* expression (*P*=5×10^−3^ and 1×10^−7^, respectively; Fig. 2c). Signature 13 mutations remain positively correlated in both sky and clouds.

### Sky and clouds show distinct mutations patterns

**Fig. 3:**
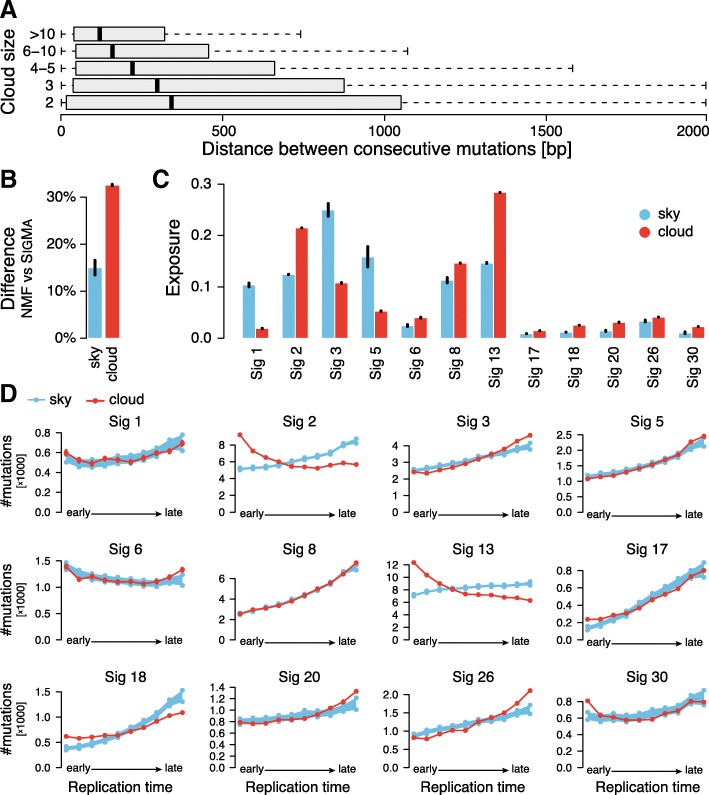
**a** Distribution of distance between consecutive mutations in clouds of various sizes (number of mutations in a cloud). **b** Difference between NMF and SIGMA in mutation signatures assigned to mutations is higher for cloud mutations. **c** Comparison of exposure to mutation signatures in sky and cloud regions based on SIGMA signature assignments. **d** Frequency distribution of the 12 mutation signatures (assigned by SIGMA) over replication time. The red line is the distribution over replication time from early to late for mutations in clouds. The blue line is the distribution of trends for sky mutations downsampled to the number of mutations found in clouds. The sampling was repeated 1000 times, and the 95% confidence intervals of the downsampled sky mutation frequencies are shown. All results show mean values with standard error of the mean (small vertical bars) from 31 random initializations of SIGMA

In SIGMA, clouds are defined as dense groups of mutations, but unlike the definition of clustered mutations [21] or processive groups [19], we make no restriction on consecutive mutations being of the same type and/or being on the same strand. We also do not require that the number of mutations in a cloud is large or filter out nearby mutations. Despite our liberal 2000-bp cutoff for maximal distance of two constitutive mutations in a cloud, median distances between mutations in the same cloud are less than 500 bp independently of its size (number of mutations in a cloud; see Fig. 3a) while the median distance between mutations in the sky is more than 150,000 bp. As expected, the differences in mutation assignments between SIGMA and NMF are much higher for the mutations that belong to clouds than to sky (Fig. 3b).

Interestingly, clouds and sky show quite different distribution of signature exposures, even though they have similar nucleotide and trinucleotide content (Additional file 1: Figure S3). For example, clouds are strongly enriched in signatures 2, 13, 18, 21, and 30 (lo*g*_2_ fold change >0.75) but depleted in signatures 1, 3, and 5 (Fig. 3c).

The above observations suggest that the properties of clouds and sky are quite different. Moreover, we observed that sky mutations show a gradual increase of mutations toward late replication regions (67% total increase), while cloud mutations show an increase towards both early and late replication regions (27% and 34% increase with respect to the lowest level, respectively; see Additional file 1: Figure S4). Therefore, we analyzed the distribution of mutations assigned to individual signatures with respect to replication time considering clouds and sky as two potentially different subpopulations. With the exception of mismatch repair signature 6, all signatures within sky are enriched in late replication regions (Fig. 3d). Some signatures, such as signatures 1, 5, and 8, show no appreciable differences in the trends between sky and clouds; however, many other signatures do. The most striking difference in the trends is displayed by the APOBEC signatures 2 and 13. Previous studies that analyzed the relation of APOBEC with replication time appeared to be contradictory. Kazaonov et al. [34] reported enrichment of APOBEC mutations in early-replicating regions and hypothesized that this unusual mutagenesis profile may be associated with a higher propensity to form single-strand DNA substrates for APOBEC enzymes in early-replicating regions. However, Morganella et al. [19] found that signature 2 is enriched in late-replicating regions suggesting that APOBEC mutations assigned to signature 2 are more efficiently repaired in early-replicating regions. They were also surprised to find that signature 13 differed from signature 2 and showed no dependency of mutation frequency on replication time (see also Additional file 1: Figure S5). Our analysis reconciles these two results and demonstrates that while APOBEC mutations associated with clouds show properties consistent with these reported by Kazaonov et al., the sky associated ones show the usual enrichment in late-replicating regions. The cumulative mutation profile depends on the individual characteristics of the sky-associated and cloud-associated subpopulations and their relative abundance. Interestingly, the proportion of cloud-associated mutations relative to sky-associated mutations is higher for signature 13 than for signature 2 (Fig. 3c) contributing to the differences in cumulative trends of these two signatures reported by Morganella et al. (Additional file 1: Figure S5).

We performed a similar analysis of signature exposures considering genomic location of mutations with respect to promoter, intragenic, and intergenic regions (Additional file 1: Figure S6). We found interesting differences between exposure distributions in sky and clouds, most of which can be explained by known biology. For example, replication origins are known to be enriched in promoters while gene poor regions are known to replicate late. This observation and the association of APOBEC signatures in clouds with early replication (Fig. 3d) can explain the high proportion of these signatures in the promoter regions while the proportion in intergenic regions is the lowest.

Overall, these analyses demonstrate that some signatures have very different properties when considered in the context of clouds versus sky, suggesting that the interplay of mutational processes that underlines the same signature in sky and in clouds might be different.

### Transition probabilities reveal associations between signatures

**Fig. 4:**
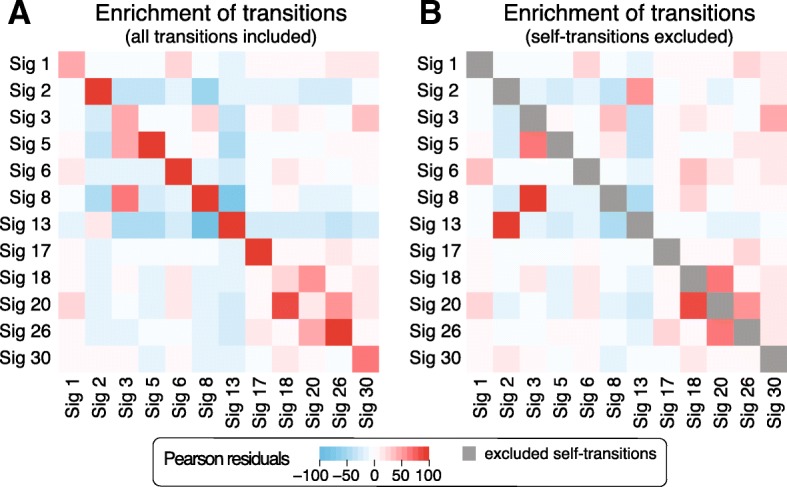
Enrichment of transition frequencies between mutation signatures in sequence-dependent cloud segments across all samples. **a** Enrichment represented as Pearson residuals between observed and expected signature frequencies shows a strong enrichment of self-transitions. **b** Enrichment computed in the same way but ignoring self-transitions to correctly estimate the enrichment of transitions between different signatures while accounting for the enrichment for self-transitions. Mean values of enrichment from random initializations of SIGMA are shown

Next, we asked if the transition probabilities can provide additional insights into the etiology of mutation signatures. Since the number of cloud mutations in individual patients is small, we used cumulative transition probabilities obtained by counting the transitions between signatures in clouds across all samples. We quantified the enrichment of transition probabilities between signatures using Pearson residuals. The most frequent transitions are from each signature to itself (Fig. 4a). Correcting for this enrichment, we then considered the enrichment between pairs of different signatures (Fig. 4b). Ten pairs of signatures showed Pearson residuals above 10 in both transition directions and are discussed below.

Expectantly, we observed an enrichment of transitions between the two APOBEC signatures 2 and 13 and between the mismatch repair signatures 6, 20, and 26. These are groups of different (and dissimilar) signatures that are known to be underlined by the same general mutagenic processes and are often found in the same samples. Interestingly, there is also a strong association between signatures 3 and 8 suggesting a relation between signature 8 and homologous recombination deficiency that was shown to underlie signature 3 [35] and is consistent with the findings of [24].

We also observed an enrichment in the transitions between signatures 18 and 30 suggesting a possible relation between these less understood signatures. Further supporting this relationship, we found that these signatures significantly co-occur in the same patients (*P*<2.2×10^−16^ for clouds based on the Fisher exact test where signatures with exposure at least 0.01 are considered to be present; co-occurrence is not significant in sky). Previous studies linked a new signature that is very similar to signature 18 to bialleic deactivation of *MUTYH*, which is involved in the base excision repair in response to oxidative damage [36–38]. Specifically, *MUTYH* is involved in repairing the damage caused by 8-oxoguanine—one of the most common DNA lesions resulting from the presence of reactive oxygen species (ROS). If not corrected, it leads to G-to-T transversion. Recent studies provided further support for the relation of signature 18 and ROS [39].

As for signature 30, recent studies linked it to mutations in the *NTHL1* gene [12]. Similarly to the *MUTYH* gene, *NTHL1* is a glycosylase that is also involved in the repair of oxidative DNA damage. Unlike *MUTYH* which is involved in the repair of oxidized purines, *NTHL1* is involved in the removal of oxidative pyrimidine lesions. If not corrected, oxidized, deaminated cytosines are a source of C-to-T transitions in vivo [40] which is consistent with the mutational profile of signature 30.

Finally, we also observed enriched transitions between signature 18 and the DNA mismatch repair (MMR) signature 20. This is consistent with the growing understanding that the MMR pathway is also important for the response to oxidative damage. In fact, mismatch repair-deficient mice show susceptibility to oxidative stress-induced intestinal carcinogenesis [41]. In addition, a study by Colussi et al. [42] showed that baseline 8-oxoG levels were higher in DNA extracted from *MSH2*- and *MLH1*-deficient cell lines. The relations between the remaining signature pairs with value of Pearson residuals above 10—(6,1), (17,26) and (8,30)—remain to be investigated.

These observations indicate that the analysis of transition probabilities can be extremely valuable in shedding light on the etiology of less understood signatures.

### Evaluation against clinical and demographic data

To show the utility of our model in the clinical setting, we evaluated the assignment of mutations to their underlying signatures using clinical and demographic data. Our analysis is based on the intuition that more accurate assignments will have higher correlation with clinical and demographic data, since multiple signatures have been shown to correspond to exogenous factors such as the patient’s age at diagnosis [17].

**Fig. 5:**
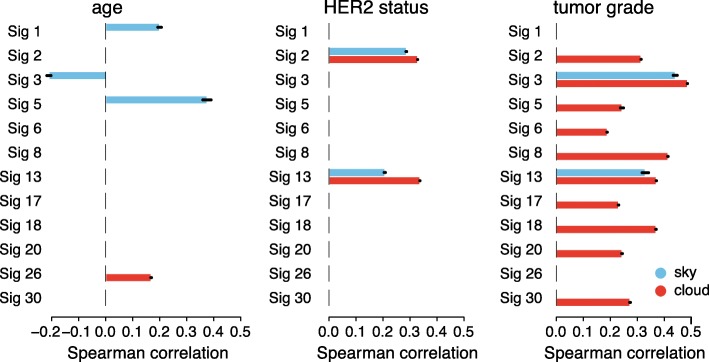
Spearman correlation coefficients between demographic or clinical features and mutations attributed to each signature in sky and cloud regions. Only significant correlations with a *p*-value cutoff of 0.001 are shown. Barplots show mean correlations with standard error of the mean (small black bars) from 31 random initializations of SIGMA

We analyzed the Spearman correlation between the number of mutations attributed to each of the signatures and five different clinical/demographic features: age, tumor grade, final estrogen-receptor (ER), progesterone-receptor (PR), and HER2 status (Fig. 5 and Additional file 1: Figure S7). Importantly, we separated mutations with respect to sky and clouds. This allows us to isolate clinical features that are correlated with patient mutations in clouds from those in sky. First, we evaluated the signatures with known etiologies that match our clinical dataset. For example, signatures 1 and 5 have been hypothesized to be active in normal cells and “clock-like” due to their correlation with the age of the patient [17]. Reassuringly, we found statistically significant association between the number of mutations attributed to signatures 1 and 5 and age and only found these correlations for mutations in sky (especially for signature 5 whose correlation with age is much stronger than previously reported in BRCA [17]). As another example, signatures 2 and 13 display patterns of mutations linked to APOBEC proteins and are correlated with APOBEC activity, which has been linked to *HER2* expression in breast cancers [43–45]. Specifically, HER2 signaling has been shown to elevate DNA replicative stress which, in turn, causes an overall increase in single-stranded DNA during replication [46] increasing opportunity for APOBEC mutations and might also induce APOBEC expression [45]. Our results also capture this relationship, with statistically significant associations between mutations attributed to both APOBEC signatures and HER2 status in both sky and clouds.

We also found significant associations of cloud mutations in most of the signatures with tumor grade. The numerous associations between cloud mutations and tumor grade might be driven in part by the general association of high-grade tumors with mutation burden (Spearman correlation of 0.48 for sky and 0.49 for clouds) as non-random distribution of mutations in highly mutated genomes can lead to emergence of clouds. Interestingly, in contrast to mutation enrichment in clouds, the increased mutation burden in sky can be attributed mostly to two signatures: 3 and 13. Since signature 3 is associated with HRD [35], which leads to defective DNA double-strand break repair, this might be an additional reason for the observed enrichment of cloud mutations [47–49]. Interestingly, only sky-associated mutations of signature 13 but not signature 2 show correlation with tumor grade. This is consistent with the recent finding of the induction of *APOBEC3B* in response to DSB [50]. In addition, previous studies demonstrated a relation between increased *APOBEC3B* enzymatic activity and tumor grade [44]. Consistently, as shown in Fig. 2c, *APOBEC3B* expression correlates with sky mutations attributed to signature 13 but not to signature 2. We report additional significant correlations for final ER and PR status in Additional file 1: Figure S7.

Finally, we compared the overall correlation of the number of mutations attributed to the 12 signatures computed with our model and NMF with the clinical and demographic data, taking the overall mutation counts into account for both models. To this end, we computed a single correlation using canonical correlation analysis (CCA) [51]. The obtained (Pearson) correlation was higher for SIGMA than NMF (0.676 vs. 0.665). These results provide further evidence that by using sequential information, SIGMA is better able to assign mutations to signatures compared to previous models.

## Discussion

In this study, we developed SIGMA, a probabilistic model of sequential dependency for mutation signatures, allowing for an accurate assignment of mutations to signatures. Application of SIGMA revealed new insights into the mutagenic processes in cancer.

Our analysis reinforced the idea that cloud (close-by) mutations have distinct properties from sky (isolated) mutations in terms of signature exposures (Fig. 3c), biological correlates (Figs. 2b, c and 3d), and clinical correlates (Fig. 5). While some of the differences between these two mutation groups have been appreciated before, e.g., [21], our analyses bring novel insights. Interestingly, mutations that are assigned to the same signatures can have distinct properties when localized in clouds versus sky suggesting that they correspond to different subpopulations. These subpopulations, despite being assigned the same signature, might correspond to different combinations of causes. As a case in point, we found that APOBEC-associated mutations have different properties with respect to replication time depending on their assignment to sky versus clouds.

To verify that our categorization of sky mutations does not suffer from a bias toward mutations in difficult-to-map regions, we downloaded regions of low mappability from [52, 53], which were previously used for mutation signature studies [21, 54]. We find that only 4523 of 3.4 million mutations (approximately 0.1%) fall in these difficult-to-map regions and conclude that this will not lead to a systematic bias that could change the conclusions of our study. Future research could further examine the partition to sky and clouds, potentially introducing a complete generative model that accounts also for the distances between mutations.

While evaluating the predictions of SIGMA using clinical and demographic data, we found a statistically significant anti-correlation of signature 3 activity (associated with homologous recombination repair deficiency [HRD]) and patient age. We hypothesize that this is in part a consequence of germline variants predisposing to HRD (such as *BRCA1* mutations; see [55]) leading to earlier onset of breast cancer. In fact, the correlation between signature 3 activity and age drops from − 0.22 to − 0.13 when removing patients with *BRCA1* or *BRCA2* germline variants as identified by Nik-Zainal et al. [24]. Thus, in general, mutation signatures whose activity is anti-correlated with age may indicate that the signature’s etiology includes predisposing germline variants.

The basic HMM model presented here can be extended and refined in various ways. In this work, we focused on modeling sequential dependency of previously validated mutational signatures from COSMIC [11]. One extension to our model, in case no prior knowledge on relevant mutation signatures is available, is to learn signatures and transitions simultaneously across multiple samples. Another possible refinement is to cast it in a Bayesian framework and add prior distributions to the model parameters. This refinement will be especially important when training the model on different cancer types where the number of samples is low.

## Conclusions

We presented the first probabilistic model of sequential dependency for mutation signatures, SIGMA. We first showed that models of sequential dependency of mutation signatures have greater predictive power for held-out data than models that ignore this dependency. Next, we found that by modeling sequential dependencies previously observed among mutations [18–21], we improved the estimation of mutation-to-signature assignment and revealed new insights into the genomic factors that bias mutational process activity. In particular, our analysis reconciled two apparently contradictory results showing that while APOBEC mutations associated with clouds show properties consistent with these reported by Kazaonov et al. [34], the sky-associated ones show the usual enrichment in late-replicating regions as observed by Morganella et al. [19]. The results obtained with SIGMA shed also new light on the etiology of signatures 18 and 30.

The ability to correctly determine which mutational processes generated a specific mutation is of primary importance for understanding of the emergence of tumors. For example, previous studies provided evidence that APOBEC activity is responsible for the generation of helical domain hotspot mutations in the *PIK3CA* gene in papilloma virus-driven tumors [56]. Computational tools like SIGMA provide the means for finding such relationships between mutational processes and gene-level cancer drivers. A more precise assignment of mutations to signatures also allows for a more precise estimation of signature exposures and, consequently, can help to uncover relations between mutational processes and clinical and demographical phenotypes that might be difficult to infer if the signature exposure is low and signature assignment noisy.

## Additional file

Additional file 1 Supplemental figures S1, S2, S3, S4, S5, S6, and S7. (PDF 226 kb)

## Data Availability

The datasets used for and generated during this study are linked to and/or available at https://github.com/lrgr/sigma.

## Abbreviations

BRCA: Breast cancer
CLLE: Chronic lymphocytic leukemia
HMM: Hidden Markov model
HRD: Homologous recombination repair deficiency
ICGC: International Cancer Genome Consortium
MMM: Multinomial mixture model. MALY: Malignant lymphoma
NMF: Non-negative matrix factorization
PACA: Pancreatic cancer
SigMa: Signature Markov model
