# Supplementary material for: Hidden Markov models lead to higher resolution maps of mutation signature activity in cancer
Source: Genome Med. 2019 Jul 26;11:49. doi: 10.1186/s13073-019-0659-1 (PMC6660659; doi:10.1186/s13073-019-0659-1)
Supplement: Supplementary file 1 — Supplemental figures S1, S2, S3, S4, S5, S6, and S7. (PDF 226 kb) [file 13073_2019_659_MOESM1_ESM.pdf]

Additional File 1 – Supplemental Figures

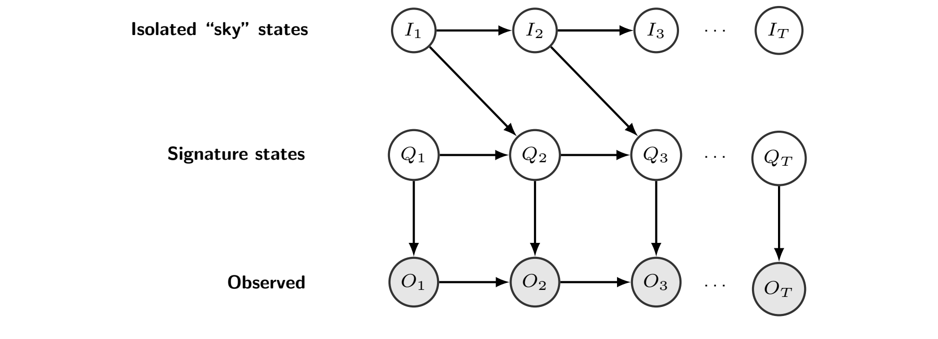

Figure S1 The dynamic Bayesian network component of the SigMa model for mutation sequences.

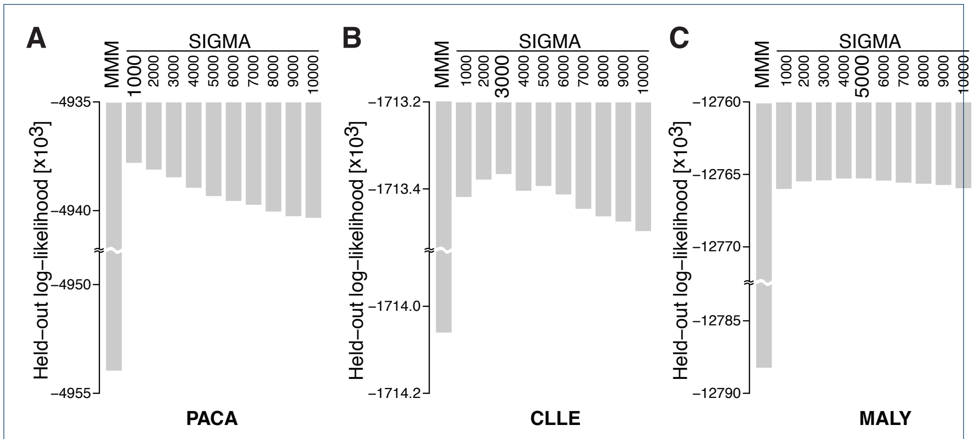

Figure S2 Comparative assessment of model performance on held-out data from three cancer types for MMM and SigMa across different distance thresholds: (**A**) Pancreatic cancer (PACA), (**B**) Chronic lymphocytic leukemia (CLLE), (**C**) Malignant lymphoma (MALY). The best performance is shown for SigMa at thresholds of 1000 bp, 3000 bp and 5000 bp for PACA, CLLE, and MALY, respectively, for which the held-out log-likelihoods are maximized. The y-axis has a customized scale with a scale break.

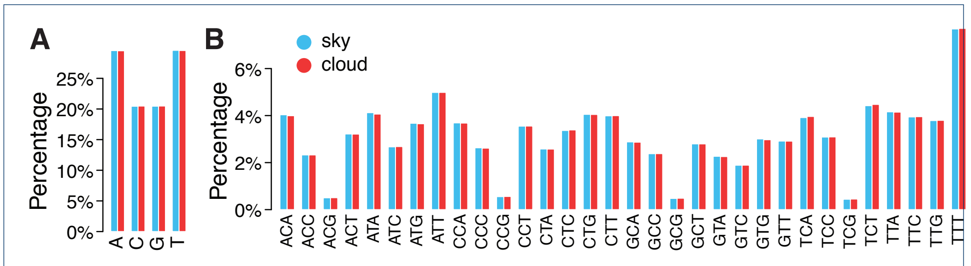

Figure S3 Nucleotide (**A**) and trinucleotide (**B**) content in "sky" and "cloud" regions. "Cloud" regions contain all genomic positions localized in cloud in at least one patient in the ICGC breast cancer dataset (2.8% of the genome), and "sky" regions consist of remaining genomic positions.

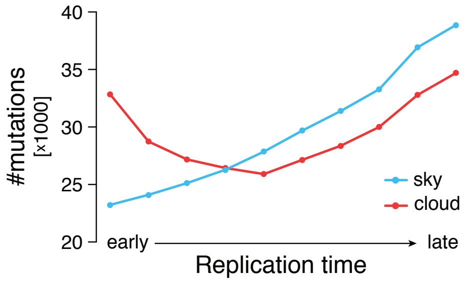

Figure S4 Comparison of the frequency distribution of mutations in sky and clouds over replication time. The counts of mutations in sky were scaled (multiplied by 0.085) to match the number of cloud mutations. The y-axis has a customized scale.

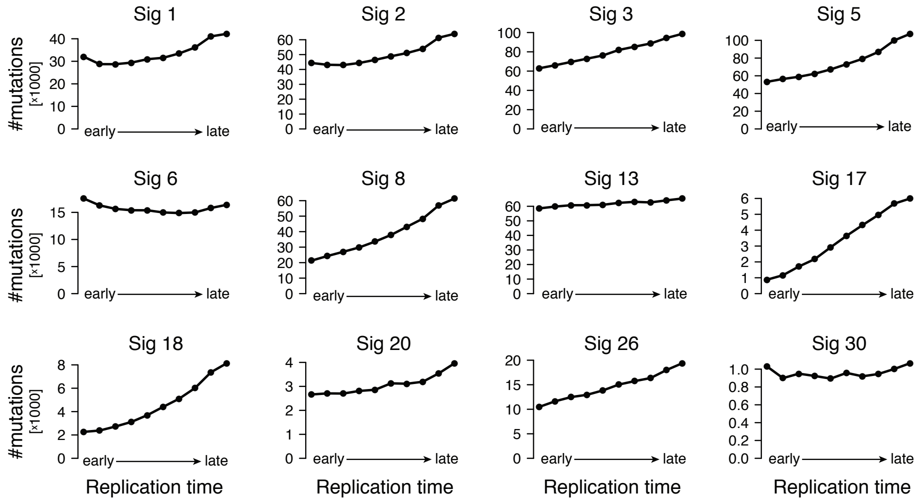

Figure S5 Frequency distribution of the 12 mutation signatures, as assigned by NMF, over replication time. The NMF signature assignments and the replication time estimates comes from Morganella et al. [19]. Mutations from both sky and clouds were analyzed together.

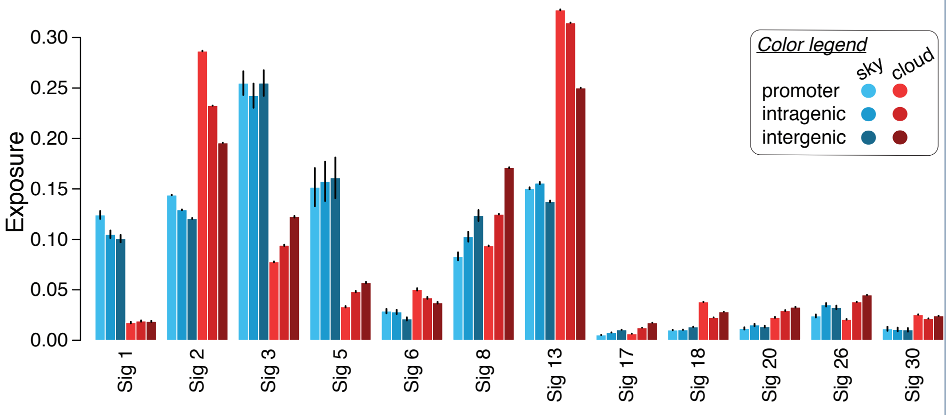

Figure S6 Comparison between exposures of mutation signatures in promoter, intragenic and intergenic regions for sky and cloud mutations based on SigMa signature assignments. Barplots show mean exposures with standard error of the mean (small black bars) from 31 random initializations of SigMa. Promoter region is a 1 kb region upstream of transcription start of a protein-coding gene, intragenic - region between transcription start and end of any annotated gene, and intergenic - region at least 10 kb away of any annotated gene.

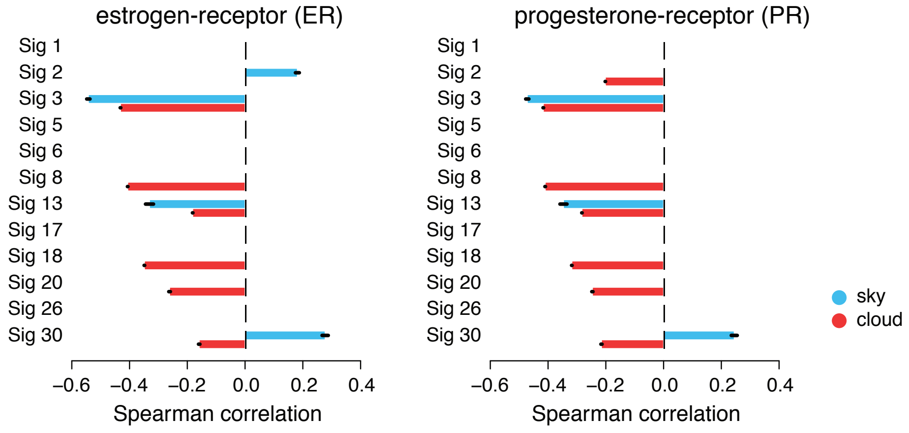

Figure S7 Spearman correlations between clinical features and mutation counts attributed to each signature in sky and cloud regions. Only significant correlations with a p-value cut-off of 0.001 are shown. Barplots show mean correlations with standard error of the mean (small black bars) from 31 random initializations of SigMa.Since in Breast Cancer patients with tumors that are ER/PR-positive a have lower risks of mortality after their diagnosis compared to women with ER- and/or PR-negative disease [57] thus a correlation opposite to the correlation with grade is expected.
